# Canine and ovine tick-borne pathogens in camels, Nigeria

**DOI:** 10.1016/j.vetpar.2016.08.014

**Published:** 2016-09-15

**Authors:** Vincenzo Lorusso, Michiel Wijnveld, Maria S. Latrofa, Akinyemi Fajinmi, Ayodele O. Majekodunmi, Abraham G. Dogo, Augustine C. Igweh, Domenico Otranto, Frans Jongejan, Susan C. Welburn, Kim Picozzi

**Affiliations:** a Division of Infection and Pathway Medicine, Edinburgh Medical School: Biomedical Sciences, The University of Edinburgh, Edinburgh, United Kingdom; b Utrecht Centre for Tick-borne Diseases, Utrecht University, Utrecht, The Netherlands; c Department of Veterinary Medicine, The University of Bari, Bari, Italy; d Nigerian Institute for Trypanosomiasis Research, VOM, Jos, Plateau State, Nigeria; e National Veterinary Research Institute, VOM, Jos, Plateau State, Nigeria; f Department of Veterinary Tropical Diseases, Faculty of Veterinary Medicine, University of Pretoria, South Africa

**Keywords:** *Anaplasma platys*, *Theileria ovis*, *Camelus dromedarius*, Camel, Tick-borne diseases, Nigeria

## Abstract

In April 2008, whole blood samples were collected from 36 dromedary camels in Sokoto, North-western Nigeria. Following PCR and reverse line blotting, twenty-two samples (61%) resulted positive for *Ehrlichia*/*Anaplasma* spp. and three (8%) for *Theileria*/*Babesia* spp., with three (8%) cases of co-infections being found. Both sequence and BLAST analyses identified *Ehrlichia*/*Anaplasma* spp. and *Theileria*/*Babesia* spp. positive cases as *Anaplasma platys* and *Theileria ovis*, respectively.

This is the first report of the detection of *A*. *platys* and *T*. *ovis* in camels from sub-Saharan Africa. The epidemiological relevance of this finding is enhanced by the close living of these animals with both dogs and small ruminants. The high prevalence detected for *A*. *platys* suggests a possible role of camels as carriers of this infection.

## Highlights

• First molecular survey on tick-borne pathogens in dromedary camels from Nigeria.
• First detection of *Anaplasma platys* and *Theileria ovis* in camels in Africa.
• Results suggest camels may be carriers of *Anaplasma platys* in Nigeria.

## Graphical abstract

**Figure:**
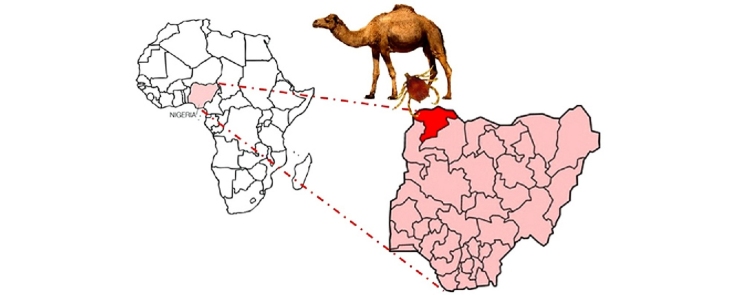

Nigeria hosts a population of approximately 20,000 dromedary camels (i.e. *Camelus dromedarius*), most of which are found in its northern States (i.e. Sokoto State) (Mohammed and Hoffmann, 2006). Camels in Nigeria are reared for milk, meat and wool production; transport; traction in agriculture; recreation and beauty pageants (Mohammed and Hoffmann, 2006). In this country, ticks and tick-borne diseases (TBDs) represent a major constraint to its livestock health and productivity (Rabana et al., 2011). In camels, heavy tick infestation is associated with anaemia, rough hair coat, retarded growth, reduction in milk production and calf mortality (Rabana et al., 2011). However, little information is currently available on the occurrence of TBDs in camels in Nigeria, with all studies published to date relying on pathogen detection through cytological examination of blood smears (Mohammed et al., 2007, Bamaiyi et al., 2011, Rabana et al., 2011). Therefore, this study aimed to assess, by molecular tools, the occurrence of tick-borne microorganisms of veterinary and zoonotic importance in dromedary camels from an area of Nigeria where camel rearing is of great economic relevance.

In April 2008, whole blood samples were collected, by jugular venipuncture, from 36 randomly selected dromedary camels (i.e. 13 males, 23 females) reared in the surroundings of the city of Sokoto (i.e. local government areas of Sokoto North, Central and South; Sokoto State), in North-western Nigeria. All sampled animals were restrained with the help of their owners and handled humanely. For each sampled animal, collected blood was applied to FTA™ cards (Whatman, BioScience, Cambridge, UK) and prepared for downstream analysis according to Ahmed et al. (2013). Based on physical examination (e.g. general somatic development and dentition) and on information provided by their owners, all sampled animals were identified as adults, being at least 5 year-old (FAO, 1990).

Reverse line blotting (RLB) targeting *Ehrlichia*/*Anaplasma* spp. and *Rickettsia* spp. 16S and *Theieleria*/*Babesia* spp. 18S partial genes were carried out as described elsewhere (Lorusso et al., 2016). Following RLB, amplicons were sequenced to confirm their identity; selected sequences amongst those obtained were deposited in GenBank on May the 15th, 2014.

Positive samples were compared according to the sex of the animals using the Fisher’s exact test with the WinPepi software. P values lower than 0.05 were considered as indicative of significance.

At RLB, 22 samples (61%) resulted positive for *Ehrlichia*/*Anaplasma* spp. ‘catch all’ probe, three (8%) for *Theileria*/*Babesia* spp., with three (8%) cases of co-infections being found. Both sequence and BLAST analyses identified *Ehrlichia*/*Anaplasma* spp. and *Theileria*/*Babesia* spp. positive samples as *Anaplasma platys* (99–100% identity with GenBank accession no. JQ894779.2 and KJ659045.1) and *Theileria ovis* (99% identity with GenBank accession no. KJ452336.1), respectively. Sequences were deposited in GenBank (GenBank accession no. KJ832066 and KJ832067 for *A*. *platys*; KJ832064 and KJ832065 for *T. ovis*).

Camels positive for *A*. *platys* infection included 7/13 (54%) males and 15/23 (65%) females, with no statistically significant difference between the two sex groups (p = 0.72). However, animals positive also for *T*. *ovis* infection included only female camels (i.e. 3/23) (Table 1).

These findings are of novelty for camels in sub-Saharan Africa (SSA).

The high positive rate (61%) of *A. platys* in camels in SSA is unexpected in that the pathogen was initially considered to be restricted to dogs, but more recently reported in cats (Lima et al., 2010), sheep (Djiba et al., 2013), cattle (Lorusso et al., 2016) and also in humans (Arraga-Alvarado et al., 2014). Moreover, DNA of anaplasmataceae closely related to *A*. *platys* was recorded in the spleen of dromedary camels from Saudi Arabia (Bastos et al., 2015). This suggests that the host range of this canine pathogen may be broader than initially considered. Nevertheless, no information is available on the pathogenicity of this microorganism in animal hosts other than dogs. Evidence indicates the role of *Rhipicephalus sanguineus* sensu lato (s.l.) ticks in the transmission of this microorganism (Ramos et al., 2014). Though preferably feeding on dogs (Dantas-Torres, 2010), *R*. *sanguineus* s.l. can also be found infesting camels as well as other livestock species (Walker et al., 2003; Lorusso et al., 2013). The high prevalence of *A. platys* infection found in this study (i.e. 61%) is seemingly attributable to the high sensitivity of the RLB method employed (Lorusso et al., 2016). It is also possible that the rather small sample size contributed to some extent to the obtainment of this prevalence. Nevertheless, the adult age of all sampled animals, as well as their provenance from different localities, suggests the possible implication of camels as ‘carriers’ of *A*. *platys* infection in this area.

*Theileria ovis* was previously detected in camels in Egypt by cytological examination of blood smears, with a prevalence of 12.6% (n = 24/190) (Mazyad and Khalaf, 2002). Interestingly, DNA from a *Theileria* sp. showing 98% of identity with *T*. *ovis* was previously detected in one dog from North-Central Nigeria (GenBank accession no. GU726904) (Kamani et al., 2013). *T*. *ovis* can be transmitted by ticks of the genera *Hyalomma* and *Rhipicephalus* (Walker et al., 2003), both possibly infesting livestock and camels in North-western Nigeria (Lorusso et al., 2013). All camel keepers from the area of sampling also owned dogs and small ruminants.

The lack of significant difference (p = 0.72) in *A*. *platys* infection rates between male and female camels suggests that sex-related differences may not influence the establishment of this infection in these animals (Kamani et al., 2008, Bamaiyi et al., 2011, Ben Said et al., 2013). Furthermore, the fact that *T*. *ovis* positive cases were recorded only in female camels, all of which were also positive for *A*. *platys*, could be related to the higher proportion of female animals compared to males sampled in the study (1.7:1) as well as to the fact that female camels, usually harboring more ticks than males (Elghali and Hassan, 2009), could have been infested by a larger number of tick specimens (i.e. *Hyalomma* spp.) responsible for the transmission of this microorganism in the study area. Due to impracticalities (i.e. lack of appropriate sampling and storing tools), this study did not include a collection or an estimation of the tick burden on the sampled animals. Nonetheless, it was noted that all camels sampled in this study were infested by ixodid ticks, including *Hyalomma* spp. and *Rhipicephalus* spp. specimens.

Future studies would therefore be advisable in order to ascertain i) the pathogenicity of *A*. *platys* and *T*. *ovis* in dromedary camels, ii) the tick species involved in their transmission to these vertebrate hosts, iii) the occurrence of these microorganisms in dogs and sheep from the study area, and thus iv) the role played by dromedary camels in the epidemiology of the canine and ovine infection respectively.

## Figures and Tables

**Table 1:** Sampled camels and results of the screening.

Camels	Sampled	Positive/Total Sampled			
		Total +	*Anaplasma platys* +	*Theileria ovis* +	*Anaplasma platys* + and *Theleria ovis* +
Males	13	7/13	7/13	0/13	0/13
Females	23	15/23	15/23	3/23	3/23
Total	36	22/36	22/36	3/36	3/36
